# The potential risks posed by inter- and intraspecies transmissions of monkeypox virus

**DOI:** 10.1080/21505594.2022.2127199

**Published:** 2022-09-24

**Authors:** Hannah Murphy, Hinh Ly

**Affiliations:** Department of Veterinary & Biomedical Sciences, College of Veterinary Medicine, University of Minnesota, Twin Cities, MN, USA

Since the 1970s, human cases of monkeypox (MPX) have been reported in 11 African countries (Benin, Cameroon, Central African Republic, the Democratic Republic of the Congo, Gabon, Cote d’Ivoire, Liberia, Nigeria, Republic of the Congo, Sierra Leone, and South Sudan), where the monkeypox virus (MPXV) is endemic [1,2]. However, in 2022, human cases of MPX have been reported in Europe (11,212 confirmed cases as of late August 2022), Canada (1,206), and the United States (16,925) from travelers, who have not traveled to any of the regions, where MPXV is endemic [1,3,4]. As the number of human cases of MPX steadily increased outside of these endemic countries, the World Health Organization (WHO) declared it as a Public Health Emergency of International Concern (PHEIC) on 23 July 2022 [5].

While it is not entirely clear how MPXV is transmitted from human-to-human (i.e. intraspecies transmission event) in the current MPX outbreak, multiple routes are possible that include but are not necessarily limited to close contact with lesions, body fluids, and respiratory droplets of the infected individual, or during intimate or sexual contact [6–8]. Transmission can also occur vertically via the placenta to cause congenital MPX [2]. Animal-to-human transmission (i.e. inter-species, zoonotic transmission, or spillover event) of MPXV can occur via a human’s direct contact with blood, bodily fluids, or cutaneous and/or mucosal lesions of MPXV-infected animals [2].

A suspected case of human-to-dog MPXV transmission in Paris, France, in June 2022^7^ highlighted a potential for human-to-animal viral transmission. In this case, two homosexual men went to the Pitié-Salpêtrière Hospital in Paris after presenting with perianal ulcerations, vesiculopustular rashes, lethargy, headaches, and fever [7]. MPXV DNA was confirmed in the two samples taken from the men by real-time PCR [7]. Interestingly, their male Italian greyhound (dog) also presented with mucocutaneous lesions 12 d after its primary owner’s symptom onset [7]. The dog tested positive for MPXV, and DNA sequences of both the dog and its primary owner were compared by using the state-of-the-art next-generation sequencing method, which showed 100% viral sequence homology of the 19.5 kilobase pairs sequence between the two samples [7]. The 100% sequence homology and the kinetics of symptom onset in the patients and subsequently in their dog strongly implicates a human-to-animal transmission event of MPXV [7].

MPXV is an orthopoxvirus of the family *poxviridae*. Orthopoxviruses are known to infect humans and animals and then back again [9–11]. The first known outbreak of MPXV outside of Africa was dated back in 2003 in the United States where pet prairie dogs were infected when they were co-housed with Gambian pouched rats and dormice (i.e. inter-species transmissions) imported from Ghana and subsequently led to over 70 human cases of MPXV [2] (i.e. zoonotic transmissions). It has been shown that several different species of rodents (wild and domestic rodents, such as prairie dogs, squirrels, marmots, groundhogs, chinchillas, giant-pouched rats, and possibly guinea pigs, mice, and rats), hedgehogs, and possibly adult rabbits can be infected (experimentally and naturally) with MPXV, and that the majority of the rodent species can also be infected with other orthopoxviruses (e.g. smallpox, cowpox, horsepox, camelpox, etc.) [10,11]. Similarly, other farmed and companion animals (e.g. cows, camels, rabbits, cats, and dogs) can also be infected by MPXV or other orthopoxviruses [10,11]. With so many animals susceptible to MPXV infection, there is clearly an increased probability for zoonotic and reverse zoonotic transmission events.

An important and unaddressed question is whether MPXV has the potential to spread into wildlife species or can become endemic in places where it has been historically absent. There are currently no known animal reservoirs for MPXV outside of Africa; however, during the 2003 outbreak of MPXV in pet prairie dogs in America, there was certainly an opportunity for a new animal reservoir to be established in a separate continent. In this event, about 300 of the 500 animals that were potentially infected with MPXV had never been found after they were distributed to various places in the United States [12]. This event had been described as a “near miss” for MPXV to establish itself in new animal species in North America [13]. The current outbreak might present another (perhaps greater) opportunity for MPXV to establish itself in new animal populations and thus could provide entirely new animal reservoir(s) for potentially more dangerous virus variants to evolve and emerge. As about half of the poxvirus’ genes in its genome encode for factors that can undermine (suppress) host immune responses to the virus infection [14,15], these factors might help explain the potential of poxviruses, including MPXV, to persist in the infected hosts and to potentially establish a productive reservoir [13], as well as their relatively large host range (i.e. the many susceptible animal species to poxviruses as described above [7–11]. Recently, a relatively small study has shown that MPXV DNA can be found in infected human fecal samples (*n* = 12) [16], which might present yet another potential mode/route for virus transmission, as some animals (e.g. rodents) could pick up the virus in the virus-laden human waste matters and thus could establish newly infected wild animal populations in traditionally non-endemic regions [11].

If the current COVID-19 pandemic has taught us anything, it is that we should develop an active surveillance effort to assess and track the prevalence and potential spread of other emerging and re-emerging viruses (and/or other human pathogens) in companion, captive (zoo), farmed, and wild animals. We and other researchers have recently provided original data showing reverse zoonoses (inter-species, human-to-animal transmissions) of SARS-CoV-2, which is the causative agent of COVID-19, in pet dogs and cats in Minnesota [17,18] and elsewhere [19,20], especially during the winter months [18] when humans were living in even closer quarters with their (infected) pets. For example, it has been shown that pets that were sleeping in the same bed with the infected human owner(s) were more likely to be associated with SARS-CoV-2 transmission [19]. It is noteworthy that the Italian greyhound reported to be infected with MPXV was also sleeping in the same bed with its infected human owner before symptom onset [7]. The parallel of MPXV and the SARS-CoV-2 virus being transmitted from human to animals via reverse zoonotic events prompts a similar need for surveillance of MPXV seropositivity in pets, as what had been done in the SARS-CoV-2 (COVID-19) pandemic in companion animals (for a review, see Murphy and Ly [20]) and wild animal species, such as white-tailed deer [20,21].

This type of an approach requires a “One Health” method that would allow for an accurate assessment of the impact that animals and the environment have had (and will continue to have) as probable means for transmission dynamics of potentially deadly pathogens into naïve human populations. This surveillance would ideally include collaboration between human and veterinary public health officials to manage domesticated, and wherever feasible, wild animals to prevent unintentional exposures of MPXV. Along with active surveillance, reverse zoonotic transmission events of pathogens also call for a plan to prevent and/or control pathogen’s spread and evolution. Toward this end, vaccine development against MPXV for vulnerable animals (as the scientific community identifies them) needs to also be considered as there are currently no orthopoxvirus vaccines approved for use in any of the known susceptible animal species [22]. By establishing a “One Health” approach for the containment of MPXV that would include surveillance and vaccination strategies, it is possible that we could effectively control the adaptive evolutionary nature of the virus to a new host species and to decrease the risk of vaccine-resistant virus strains to emerge [23], which would help decrease the risk of MPXV being established in new animal reservoir(s) and outside of the previously known endemic regions.

## Data Availability

No primary data (figures and tables) are included in this article.

## References

[1] World Health Organization. Monkeypox Outbreak Europe; 2022. p. 1–11. Available from https://www.who.int/emergencies/situations/monkeypox-oubreak-2022

[2] World Health Organization. Monkeypox; 2022. Available from https://www.who.int/news-room/fact-sheets/detail/monkeypox

[3] Thornhill JP, Barkati S, Walmsley S, et al. Monkeypox virus infection in humans across 16 countries—April–June 2022. N Engl J Med. 2022;387(8):679–691. DOI:10.1056/NEJMoa2207323 35866746

[4] CDC. 2022. Monkeypox outbreak global map. Available from: https://www.cdc.gov/poxvirus/monkeypox/response/2022/world-map.html.

[5] Wenham C, Eccleston-Turner M. Monkeypox as a PHEIC: implications for global health governance. Lancet. 2022;6736:1–3. 10.1016/S0140-6736(22)01437-4 PMC9342918 35926551

[6] Brown K, Leggat PA. Human monkeypox: current state of knowledge and implications for the future. Trop Med Infect Dis. 2016;1:1–13. 10.3390/tropicalmed1010008 PMC6082047 30270859

[7] Seang S, Burrel S, Todesco E,et al. Evidence of human-to-dog transmission of monkeypox virus. *Lancet (London, England)*. 2022;6736:1–2. 10.1016/S0140-6736(22)01487-8 PMC9536767 35963267

[8] Monkeypox: How it spreads. Available from: http://www.cdc.gov/poxvirus/monkeypox/response/2022/world-map.html

[9] Macneill A. Dog that caught Monkeypox highlights risk to pets and wild animals. Sci Am. 2022.

[10] Shanley JD. Poxviruses. *Medscape*; 2022. Available from: https://emedicine.medscape.com/article/226239-overview

[11] Monkeypox in animals. *Centers for Disease Control and Prevention*. Available from: https://www.cdc.gov/poxvirus/monkeypox/veterinarian/monkeypox-in-animals.html (2022).

[12] FDA. Control of communicable diseases; restrictions on African rodents, prairie dogs, and certain other animals. interim final rule; supplement and partial reopening of comment period. *Fed Regist*. 2007;72(34):7825–7826. 17319058

[13] Cohen J. Monkeypox could establish new reservoirs in animals. Science. 2022;376(6599):1258–1259. 35709274 10.1126/science.add4868

[14] Lu Y, Zhang L. DNA-Sensing antiviral innate immunity in poxvirus infection. Front Immunol. 2020;11:1–7. 32983084 10.3389/fimmu.2020.01637 PMC7483915

[15] Smith GL, Benfield CTO, Maluquer de Motes C, et al. Vaccinia virus immune evasion: mechanisms, virulence and immunogenicity. *J Gen Virol*. 2013;94(11):2367–2392. DOI:10.1099/vir.0.055921-0 23999164

[16] Peiró-Mestres A, Fuertes I, Camprubí-Ferrer D, et al. Frequent detection of monkeypox virus DNA in saliva, semen, and other clinical samples from 12 patients, Barcelona, Spain, May to June 2022. Euro Surveill. 2022;27(28):2200503. 10.2807/1560-7917.ES.2022.27.28.2200503 PMC9284919 35837964

[17] Dileepan M, Di D, Huang Q, et al. Seroprevalence of SARS-CoV-2 (COVID-19) exposure in pet cats and dogs in Minnesota, USA. Virulence. 2021;12(1):1597–1609. DOI:10.1080/21505594.2021.1936433 34125647 PMC8205054

[18] Murphy H, Sanchez S, Ahmed S, et al. SARS-CoV-2 in companion animals: do levels of SARS-CoV-2 seroconversion in pets correlate with those of pet’s owners and with protection against subsequent SARS-CoV-2 infection? Virulence. 2022;13(1):1216–1220. DOI:10.1080/21505594.2022.2098922 35799426 PMC9345533

[19] Calvet GA, Pereira SA, Ogrzewalska M, et al. Investigation of SARS-CoV-2 infection in dogs and cats of humans diagnosed with COVID-19 in Rio de Janeiro, Brazil. PLoS One. 2021;16(4):1–21. DOI:10.1371/journal.pone.0250853 PMC8081175 33909706

[20] Murphy HL, Ly H. Understanding the prevalence of SARS-CoV-2 (COVID-19) exposure in companion, captive, wild, and farmed animals. Virulence. 2021;12(1):2777–2786. 34696707 10.1080/21505594.2021.1996519 PMC8667879

[21] USDA. Questions and answers : results of study on SARS-CoV-2 in white-tailed deer; 2021. Available from: https://www.aphis.usda.gov/animal_health/one_health/downloads/qa-covid-white-tailed-deer-study.pdf.

[22] Monkeypox. *American Veterinary Medical Association*; 2022. Available from: https://www.avma.org/resources-tools/one-health/veterinarians-and-public-health/monkeypox.

[23] van Oosterhout C, Stephenson JF, Weimer B, et al. COVID-19 adaptive evolution during the pandemic – implications of new SARS-CoV-2 variants on public health policies. Virulence. 2021;12(1):2013–2016. DOI:10.1080/21505594.2021.1960109 34320912 PMC8330993

